# Japanese Gastric Cancer Treatment Guidelines 2021 (6th edition)

**DOI:** 10.1007/s10120-022-01331-8

**Published:** 2022-11-07

**Authors:** Japanese Gastric Cancer Association

**Affiliations:** Japanese Gastric Cancer Association, 465 Kajii-cho, Kawaramachihirokoji, Kamigyo-ku, Kyoto, 602-0841 Japan

**Keywords:** Treatment guidelines, Surgery, Endoscopic resection, Chemotherapy, Evidence based

## Abstract

The sixth edition of the Japanese Gastric Cancer Treatment Guidelines was completed in July 2021, incorporating new evidence that emerged after publication of the previous edition. It consists of a text-based “Treatments” part and a “Clinical Questions” part including recommendations and explanations for clinical questions. The treatments parts include a comprehensive description regarding surgery, endoscopic resection and chemotherapy for gastric cancer. The clinical question part is based on the literature search and evaluation by an independent systematic review team. Consequently, not only evidence for each therapeutic recommendation was clearly shown, but it also identified the research fields that require further evaluation to provide appropriate recommendations.

## Preface to the English version

This English version is based on the Japanese version of the Japanese Gastric Cancer Treatment Guidelines, published as a book in 2021. However, this version reflects some of the new evidence that emerged since the publication of the Japanese version.

## Preface to the Japanese Gastric Cancer Treatment Guidelines 6th edition

The sixth edition of the Japanese Gastric Cancer Treatment Guidelines was completed in July 2021, incorporating new evidence that emerged after publication of the previous edition. Information on some of the new evidence had already been published as quick bulletins on the website of the Japanese Gastric Cancer Association (JGCA) to supplement the previous edition.

Prior to the initiation of the editing process, the concept and style of the new edition were reconsidered by the committee members. To serve as an easy-to-use guideline in clinical practice, it was then decided that the sixth edition would follow the same format as the previous version. Namely, the sixth edition consists of a text-based “Treatments” part and a “Clinical Questions” part including recommendations and explanations for clinical questions.

To promote evidence-based medicine, besides adhering to the philosophy of our senior members who compiled the first edition, which was the first of the cancer guidelines issued in Japan, the current committee members used the method recommended by the Medical Information Network Distribution Service (MINDS), which has established a clear definition of guidelines and created and publicized a standard methodology for their compilation. Thus, the committee members proposed several relevant Clinical Questions (CQs), an independent systematic review team searched and evaluated the literature, and then the committee members decided the strength of each recommendation based on the review results by consensus. Consequently, not only was evidence for each therapeutic recommendation clearly shown, the research fields that require further evaluation to provide appropriate recommendations were identified.

The major points of revision in the current edition are listed below:

1. The number of CQs has increased to 32 items, including surgery, endoscopic therapy, chemotherapy, and palliative therapy.
2. Based on the results of the prospective study jointly conducted by JGCA and the Japan Esophageal Society, an algorithm of the surgical approach and lymph node dissection for cT2–T4 has been shown. Additionally, three CQs for esophagogastric junctional carcinoma have been proposed, and recommendations for them are provided.
3. Recommendations for laparoscopic surgery and robot-assisted surgery based on the latest research results were provided.
4. Chemotherapeutic regimens to treat unresectable advanced or recurrent gastric cancer patients have been classified into either the “recommended regimens” or “conditionally recommended regimens” as algorithms in the “Treatments” part. The evidence level as defined in the MINDS manual ver. 2 was provided for all regimens classified into the “recommended regimens” category. Though treatment options increased, evidences generated by direct comparisons between each regimen do not exist. Therefore, no priorities have been given, and evidence levels have not been listed.
5. The latest research results of immune checkpoint inhibitors have been explained in CQ23.

## Treatments

### Treatment modalities and their indications

#### Algorithm of standard treatments to be recommended in clinical practice

**Fig. 1:**
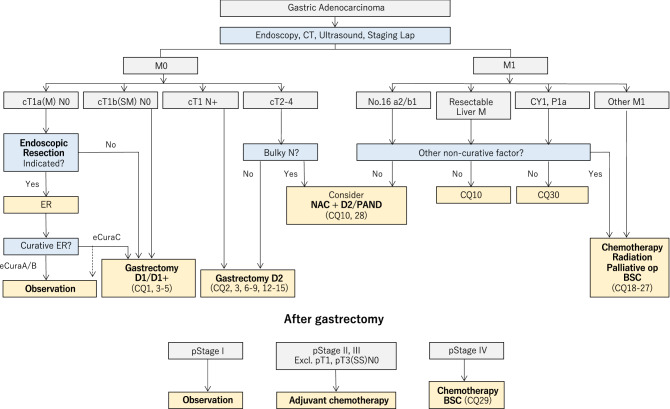
Algorithm of standard treatments. T/N/M and Stage are used in conjunction with the Japanese Classification of Gastric Carcinoma 15th edition [1] and TNM classification 8th edition [2]

The algorithm is shown in Fig. 1. Description of the tumor status (T/N/M and stage) in this edition is based on the 15th edition of the Japanese Classification of Gastric Carcinoma [1], which is identical to the 8th edition of the International Union Against Cancer (UICC)/TNM Classification [2].

#### Summary of T, N, and M categories and stage grouping based on the 15th edition of the Japanese Classification of Gastric Carcinoma [1]

N grade is categorized according to the number of metastatic lymph nodes among the regional lymph nodes (No. 1–12. 14v); N1: 1–2, N2: 3–6, N3a: 7–15, N3b: ≥ 16.

M1: metastasis outside the regional lymph nodes (including CY1).

Stage grouping: See Table 1.

**Table 1:** Stage grouping

		M0		M1
		N0	N(+)	any N
Clinical stages (cTNM, cStage, to be decided based on preoperative imaging, staging laparoscopy findings and intraoperative findings)				
T1 (M, Sm)/T2 (MP)		I	IIA	IVB
T3 (SS)/T4a (SE)		IIB	III	
T4b (SI)		IVA		

	M0					M1
	N0	N1	N2	N3a	N3b	Any N
Pathological stages (pTNM, PStage, to be decided based on pathologic findings of the resected specimen)						
T1a (M)/pT1b (SM)	IA	IB	IIA	IIB	IIIB	IV
T2 (MP)	IB	IIA	IIB	IIIA	IIIB	
T3 (SS)	IIA	IIB	IIIA	IIIB	IIIC	
T4a (S)	IIB	IIIA	IIIA	IIIB	IIIC	
T4b (SI)	IIIA	IIIB	IIIB	IIIC	IIIC	

## Surgery

### Types and definitions of gastric surgery

#### Standard gastrectomy and non-standard gastrectomy in surgery with curative intent

##### Standard gastrectomy

**Fig. 2:**
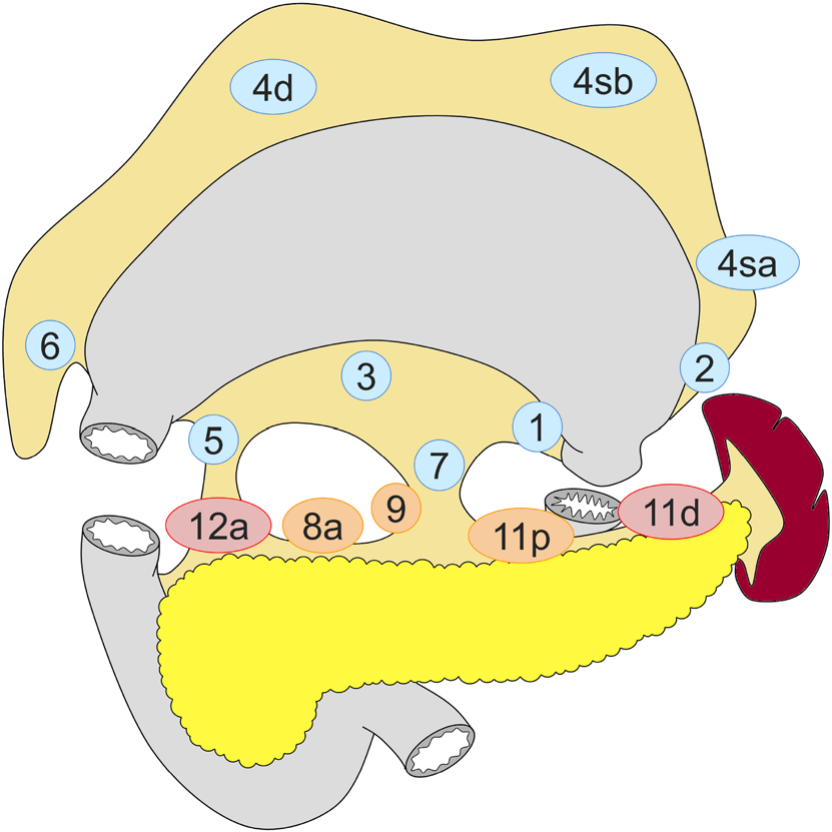
Lymph node dissection in total gastrectomy. Lymph node stations in blue need to be dissected in D1 dissection. In addition, lymph node stations in orange need to be dissected in D1 + dissection and lymph node stations in red as well in D2 dissection

Standard gastrectomy is the principal surgical procedure performed with curative intent. It involves resection of at least two-thirds of the stomach with a D2 lymph node dissection (refer to the section of “Lymph node dissection” and Figs. 2 and 5 for the definition of D-categories).

##### Non-standard gastrectomy

In non-standard gastrectomy, the extent of gastric resection and/or lymphadenectomy is determined depending on tumor stage. It includes modified surgery and extended surgery.

##### Modified surgery

The extent of gastric resection and/or lymphadenectomy is reduced (D1, D1 + , etc.) compared to standard surgery.

##### Extended surgery

1) Gastrectomy with combined resection of adjacent involved organs. 2) Gastrectomy with extended lymphadenectomy exceeding D2.

#### Non-curative surgery

Non-curative surgery is offered to patients who are considered incurable. It can be semi-classified into either palliative surgery or reduction surgery depending on the aim of surgery.

##### Palliative surgery

Serious symptoms such as bleeding or obstruction may develop in a patient with advanced/metastatic gastric cancer. Surgery to relieve imminent symptoms may then be considered an option, and palliative gastrectomy or gastrojejunostomy is selected depending on the resectability of the primary tumor and/or surgical risks. Surgical intervention for cases with gastric outlet obstruction has been reported to result in maintaining quality of life (QOL), improvement of oral intake [3], and a good prognosis, especially in cases with maintenance of good QOL [4].

##### Reduction surgery

Reduction surgery is defined as gastrectomy performed for patients with incurable factors such as unresectable liver metastasis and peritoneal metastasis, while suffering from no tumor-associated symptoms such as bleeding, obstruction, and pain. It aims to prolong survival or to delay the onset of symptoms by reducing tumor volume. However, an international, cooperative, randomized, controlled trial (REGATTA, JCOG0705/KGCA01) failed to prove a survival benefit of reduction surgery [5]. Therefore, surgeons are strongly advised not to perform this type of surgery for a patient for whom systemic chemotherapy is appropriate (CQ1).

### Extent of gastric resection

#### Surgery for gastric cancer

Surgery for gastric cancer is defined as follows in the order of the stomach volume to be resected.

- Total gastrectomy: total resection of the stomach including the cardia and pylorus.
- Distal gastrectomy: stomach resection including the pylorus. The cardia is preserved. In the standard gastrectomy, two-thirds of the stomach is resected.
- Pylorus-preserving gastrectomy (PPG): stomach resection preserving the upper third of the stomach and the pylorus along with a portion of the antrum.
- Proximal gastrectomy (PG): stomach resection including the cardia (esophagogastric junction). The pylorus is preserved.
- Segmental gastrectomy: circumferential resection of the stomach preserving the cardia and pylorus.
- Local resection: non-circumferential resection of the stomach.
- Non-resectional surgery: bypass surgery, gastrostomy, jejunostomy.
  In addition, surgery for cancer of the gastric remnant is defined as follows.
- Completion gastrectomy: total resection of the remnant stomach including the cardia or pylorus depending on the type of previous gastrectomy.
- Subtotal resection of remnant stomach: distal resection of the remnant stomach preserving the cardia.

#### Determination of the extent of gastric resection

##### Resection margin

A sufficient resection margin should be ensured when determining the resection line in gastrectomy with curative intent. A proximal margin of at least 3 cm is recommended for T2 or deeper tumors with an expansive growth pattern (types 1 and 2) and 5 cm for those with an infiltrative growth pattern (types 3 and 4). When these rules cannot be satisfied, it is advisable to examine the whole thickness of the proximal resection margin by frozen section. For tumors invading the esophagus, a resection margin > 5 cm is not necessarily required, but frozen section examination of the resection line is preferable to ensure an R0 resection.

For T1 tumors, a gross resection margin of 2 cm should be obtained. When the tumor border is unclear, and difficulties in deciding on the resection line are expected, preoperative endoscopic marking of the tumor border by clips based on the biopsy results would be helpful.

##### Selection of gastrectomy

The standard surgical procedure for clinically node-positive (cN +) or T2–T4a tumors is either total or distal gastrectomy. Distal gastrectomy is selected when a satisfactory proximal resection margin (see above) can be obtained. When obtaining a clean proximal resection margin is not possible, total gastrectomy is selected. Even in a case that a satisfactory proximal resection margin can be obtained, pancreatic invasion by tumor requiring pancreaticosplenectomy necessitates total gastrectomy regardless of the tumor location. Total gastrectomy with splenectomy should be considered for tumors that are located along the greater curvature. For adenocarcinoma of the esophagogastric junction, proximal gastrectomy is also considered (CQ14).

For cT1N0 tumors, the following types of gastric resection can be considered according to tumor location.

- Pylorus-preserving gastrectomy (PPG): for tumors in the middle portion of the stomach with the distal tumor border at least 4 cm proximal from the pylorus (CQ4).
- Proximal gastrectomy: for proximal tumors where more than half of the distal stomach can be preserved.
- Local resection of the stomach and segmental gastrectomy should still be regarded as investigational treatments.

### Lymph node dissection

#### Extent of lymph node dissection

The extent of lymphadenectomy is classified by the D-level criteria into D1, D1 + , or D2, and is defined as follows according to the type of gastrectomy conducted. The indications for each of the D levels are described in the subsequent section. See the descriptions in “Esophagogastric junctional cancer” for the current recommendations on the extent of lymph node dissection for esophagogastric junctional carcinoma.

#### Definition of the D levels

The extent of systematic lymphadenectomy is defined as follows, according to the type of gastrectomy conducted. When the extent of lymphadenectomy performed does not fully comply with the D-level criteria, the lymph node station that has been additionally resected or left in situ could be recorded as in the following examples: D1 (+ No. 8a), D2 (− No. 12a). However, when entering data in the nationwide database, the D levels need to be strictly determined and should be downgraded when omitting resection of any of the lymph node stations that should have been resected to fulfill the criteria of a certain D level. (e.g., D2 (− No. 12a) should be entered as D1 +).

#### Total gastrectomy (Fig. 2)

D0: lymphadenectomy less than D1.

D1: Nos. 1–7.

D1 + : D1 + Nos. 8a, 9, 11p.

D2: D1 + Nos. 8a, 9, 11p, 11d, 12a.

For tumors invading the esophagus, resection of Nos. 19, 20, and 110* should be added to D2.

#### Distal gastrectomy (Fig. 3)

D0: lymphadenectomy less than D1.

D1: Nos. 1, 3, 4sb, 4d, 5, 6, 7.

D1+: D1 + Nos. 8a, 9.

D2: D1 + Nos. 8a, 9, 11p, 12a.

**Fig. 3:**
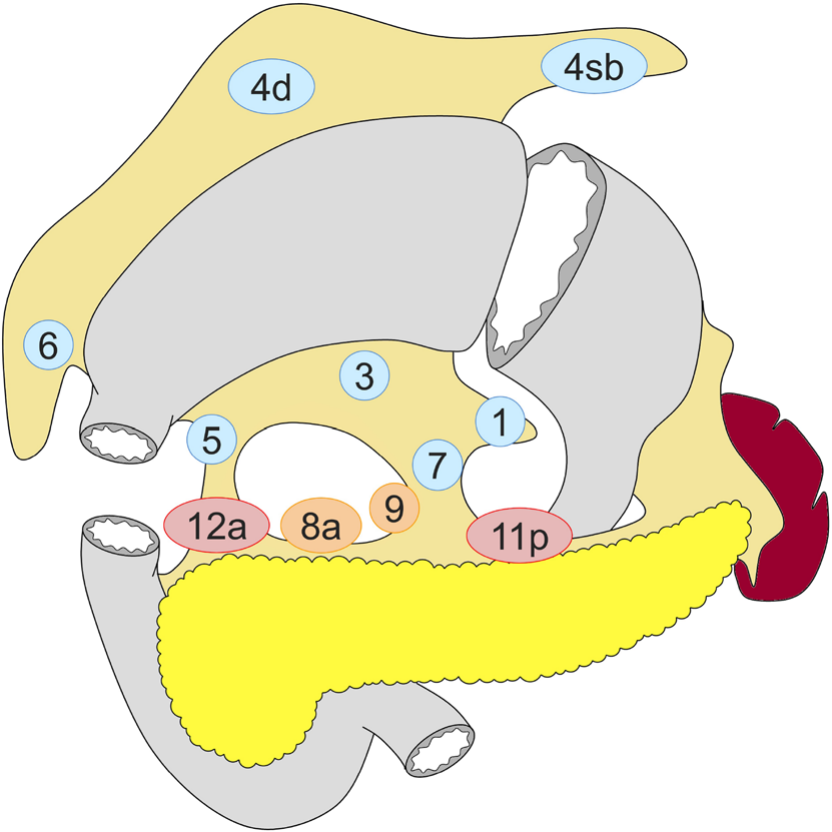
Lymph node dissection in distal gastrectomy. Lymph node stations in blue need to be dissected in D1 dissection. In addition, lymph node stations in orange need to be dissected in D1 + dissection and lymph node stations in red as well in D2 dissection

#### Pylorus-preserving gastrectomy (Fig. 4)

D0: lymphadenectomy less than D1.

D1: Nos. 1, 3, 4sb, 4d, 6**, 7.

D1+: D1 + Nos. 8a, 9.

**Fig. 4:**
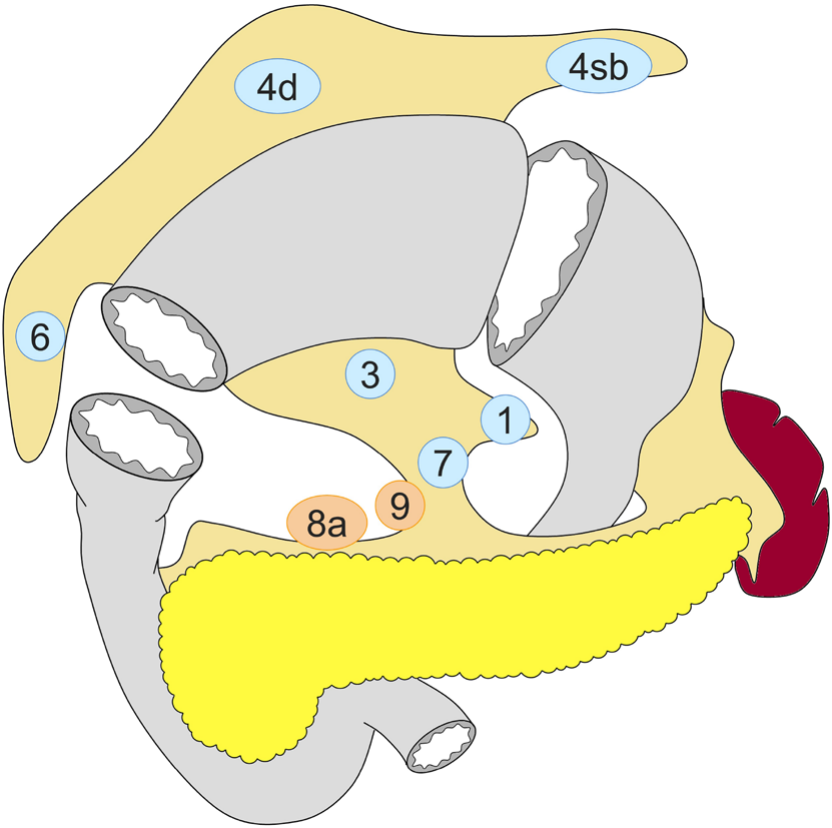
Lymph node dissection in pylorus-preserving gastrectomy. Lymph node stations in blue need to be dissected in D1 dissection. In addition, lymph node stations in orange need to be dissected in D1+ dissection

#### Proximal gastrectomy (Fig. 5)

D0: lymphadenectomy less than D1.

D1: Nos. 1, 2, 3a, 4sa, 4sb, 7

D1+: D1 + Nos. 8a, 9, 11p

D2: D1 + Nos. 8a, 9, 11p, 11d

For tumors invading the esophagus, Nos. 19, 20, and 110* should additionally be dissected in D2.

*No. 110 lymph nodes (lower thoracic para-esophageal nodes) in gastric cancer invading the esophagus are those attached to the lower part of the esophagus that is removed to obtain a sufficient resection margin.

**D level should not be changed in cases in which No. 6i was incompletely dissected in pylorus-preserving gastrectomy.

**Fig. 5:**
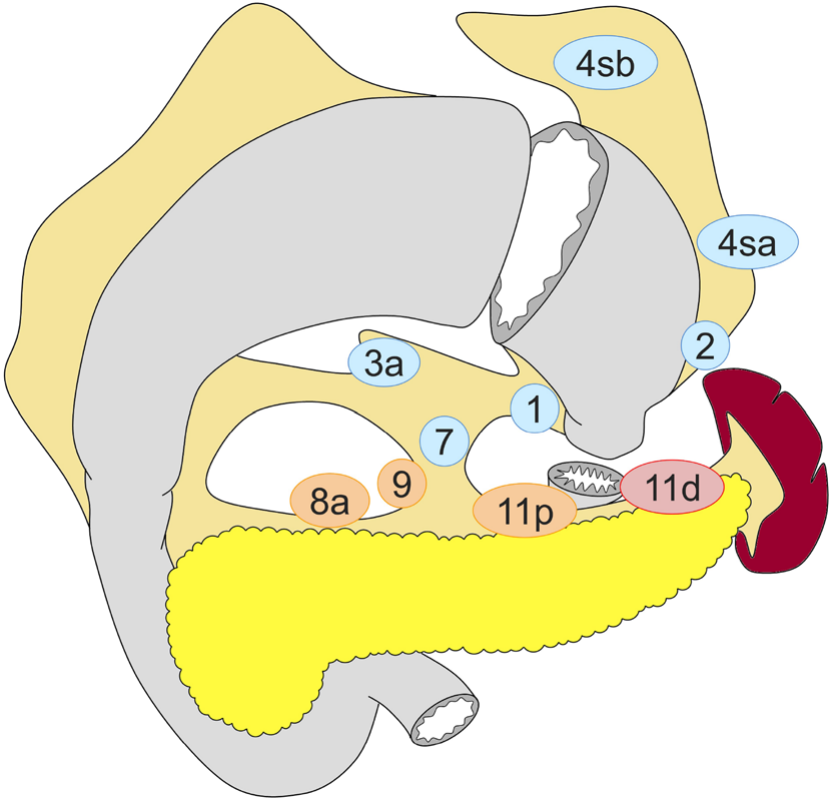
Lymph node dissection in proximal gastrectomy. Lymph node stations in blue need to be dissected in D1 dissection. In addition, lymph node stations in orange need to be dissected in D1+ dissection and lymph node stations in red as well in D2 dissection

#### Indications for lymph node dissection

In principle, D2 lymphadenectomy is indicated for cN + or ≥ cT2 tumors and a D1 or D1 + for cT1N0 tumors. Since pre- and intraoperative diagnoses regarding the depth of tumor invasion and nodal involvement remain unreliable, D2 lymphadenectomy should be performed whenever the possibility of nodal involvement cannot be dismissed.

##### D1 lymphadenectomy

D1 lymphadenectomy is indicated for cT1a tumors that do not meet the criteria for EMR/ESD, and for cT1bN0 tumors that are histologically of differentiated type and 1.5 cm or smaller in diameter.

##### D1 + lymphadenectomy

D1 + lymphadenectomy is indicated for cT1N0 tumors other than the above.

##### D2 lymphadenectomy

D2 lymphadenectomy is indicated for potentially curable cT2–T4 tumors, as well as cT1N + tumors. The spleen should be preserved in total gastrectomy for advanced cancer of the proximal stomach provided the tumor does not involve the greater curvature [6] (CQ7).

##### D2 + lymphadenectomy

Gastrectomy with extended lymphadenectomy beyond D2 is classified as a non-standard gastrectomy, and could be considered for the following cases, although hard evidence is lacking, on the condition that it can be conducted safely.

- Dissection of No. 10 (splenic hilar lymph nodes) with or without splenectomy for cancer of the proximal stomach invading the greater curvature (D2 + No. 10).
- Dissection of No. 14v (superior mesenteric venous lymph node) for cancer of the distal stomach with metastasis to the No. 6 lymph nodes (D2 + No. 14v).
- Dissection of No. 13 (posterior pancreas head lymph node) for cancer invading the duodenum (D2 + No. 13) [7]. Metastases to the No. 13 nodes, which are not included in the regional lymph nodes for gastric cancer, should usually be classified as M1. However, since the No. 13 nodes are among the regional lymph nodes for cancer of the duodenum according to the TNM classification and the Japanese Classification of Gastric Carcinoma 15th edition, these should be regarded as regional lymph nodes once gastric cancer invades the duodenum.
- Dissection of No. 16 (abdominal aortic lymph node) after neoadjuvant chemotherapy for cancer with extensive lymph node involvement (D2 + No. 16) (CQ10).

#### Esophagogastric junctional cancer

**Fig. 6:**
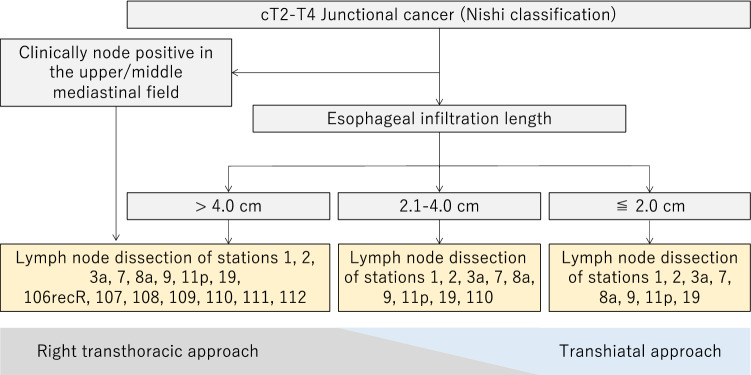
Algorithm of the surgical approach and lymph node dissection for esophagogastric junctional carcinoma

The current edition of the Japanese Gastric Cancer Treatment Guidelines defines the extent of lymphadenectomy according to the type of gastrectomy, regardless of tumor location. However, only for esophagogastric junctional cancer, either adenocarcinoma or squamous cell carcinoma, of which the center is located within 2 cm of the esophagogastric junction, there is no consensus on the type of resection and the extent of lymphadenectomy as a standard of care for this category. The Japanese Gastric Cancer Association and Japan Esophageal Society joined forces to conduct a prospective study of esophagogastric junctional cancer of cT2-T4, and the incidences of lymph node metastasis were examined [8]. It was found that the incidence of lymph node metastasis differed according to the length of esophageal invasion; a low incidence of mediastinal lymph node metastasis in tumors with esophageal invasion shorter than 2 cm, especially less than 1 cm; a high incidence of lower mediastinum lymph nodes (No.110), but a low incidence of upper and middle mediastinum lymph nodes in tumors with esophageal invasion of 2.1–4.0 cm; and a high incidence of both upper and middle mediastinum lymph nodes in tumors with esophageal invasion greater than 4 cm. Based on these results, Fig. 6 shows the approach and algorithm for dissecting regional lymph nodes with a probability of metastasis of 10% or more (Fig. 6). Although it has not been confirmed, because no survival results have been obtained, it seems reasonable to follow this algorithm for the treatment of esophageal junction carcinoma of cT2 or deeper at present.

#### Extent of the resection of the esophagus and stomach

One of the following procedures is selected for esophagogastric junctional cancer: proximal gastrectomy with or without lower esophageal resection, total gastrectomy with or without lower esophageal resection, or esophageal resection and proximal gastrectomy (CQ14).

#### Extent of lymphadenectomy (CQ12, 13)

Although long-term results for the survival benefit of lymphadenectomy following the algorithm have not yet been obtained, it seems reasonable to follow this algorithm for the treatment of esophagogastric junctional cancer of cT2 or deeper. Nevertheless, the guidelines currently do not render either more extensive or limited lymphadenectomies inappropriate. However, even in tumors with esophageal invasion of 2 cm or less, surgeons are expected to dissect a part of No. 110 lymph nodes which are attached to the portion of esophagus resected to obtain a sufficient resection margin. The definition of D level follows surgery for gastric carcinoma with esophageal invasion, but D2 includes Nos. 106recR, 107, 108, 109, 111, and 112 in tumors whose length of esophageal invasion is greater than 4 cm.

### Miscellaneous

#### Vagal nerve preservation

It has been reported that preservation of the hepatic branch of the anterior vagus and/or the celiac branch of the posterior vagus contributes to improving postoperative quality of life through reducing post-gastrectomy gallstone formation, diarrhea, and/or weight loss (CQ4).

#### Omentectomy

Removal of the greater omentum is usually integrated in the standard gastrectomy for T3 or deeper tumors. For T1/T2 tumors, the omentum more than 3 cm away from the gastroepiploic artery may be preserved (CQ6). A clinical trial confirming the non-inferiority of omentum preservation to omentectomy for T3 or deeper tumors (JCOG1711) is now underway.

#### Bursectomy

Bursectomy for tumors penetrating the serosa of the posterior gastric wall had been performed with the aim of removing microscopic tumor deposits within the omental cavity. However, the survival benefit of this procedure has been denied by a large-scale, randomized trial (JCOG1001), not only for all enrolled patients, but also for subsets with T4a tumors and tumors located in the posterior wall [9].

#### Combined resection of adjacent organ(s)

For tumors in which the primary or metastatic lesion directly invades adjacent organs, combined resection of the involved organ may be performed to obtain an R0 resection.

#### Approaches to the lower esophagus

A transhiatal abdominal approach has been recommended for gastric cancers invading less than 3 cm of the distal esophagus based on the results of JCOG9502 trial, and a transthoracic approach has been considered when a greater length of esophagus is involved [10]. The results of a recently reported cooperative study by the Japan Gastric Cancer Association and the Japanese Esophageal Society suggest that dissection of the upper and middle mediastinum might be omitted when the length of esophageal invasion is 4 cm or less. Therefore, a transhiatal abdominal approach can be recommended for cases where the length of esophageal invasion is 4 cm or less, if safe excision and reconstruction are technically possible.

#### Laparoscopic surgery

The non-inferiority of laparoscopic distal gastrectomy for clinical stage I gastric cancer to open distal gastrectomy was confirmed in phase 3, randomized, controlled, clinical trials conducted in Japan and Korea (JCOG0912, KLASS-01) [11, 12]. Therefore, laparoscopic distal gastrectomy for clinical stage I gastric cancer is strongly recommended as one of the standard treatment options. The feasibility of laparoscopy-assisted total or proximal gastrectomy has also been confirmed in a single-arm, confirmatory, clinical trial (JCOG1401) [13]. The survival data of the JCOG0912 trial can be extrapolated in total or proximal gastrectomy for clinical stage I gastric cancer. However, since survival data of the trial have not been clearly reported, laparoscopic total or proximal gastrectomy is weakly recommended in the current guidelines (CQ1). All surgical procedures must be performed by a qualified surgeon in the endoscopic surgical skill qualification system of the Japanese Society of Endoscopic Surgery or a surgeon with equivalent skills, or under the guidance of an instructor with equivalent skills.

For advanced gastric cancer, large-scale, randomized, clinical trials confirming safety and long-term survival of laparoscopic distal gastrectomy have been conducted in Japan, Korea, and China (JLSSG0901, KLASS-02, CLASS-01) [14–16]. Safety analyses showed that no increase of complications after surgery was observed with the laparoscopic approaches. The non-inferiority of overall survival of laparoscopic distal gastrectomy to open distal gastrectomy has also been confirmed in CLASS-01 [17] and KLASS-02 [18] studies. However, the estimated blood loss during laparoscopic distal gastrectomy was suppressed to the extreme (30 mL) in the JLSSG0901 trial while the operation took more than 60 min longer compared with the CLASS or KLASS trials, suggesting that details of surgical technique in laparoscopic surgery may not have been identical among the three countries. The place of laparoscopic distal gastrectomy for clinically stage II or more advanced gastric cancer will be discussed after scrutinizing results of the JLSSG0901 trial, which will be available in 2022 (CQ2).

#### Robot-assisted surgery

Robot-assisted surgery for gastric cancer was approved for health insurance coverage in 2018, and it has been adopted in many facilities as a procedure that enables more advanced surgery. In Japan, it has been reported that postoperative complications can be reduced with robot-assisted surgery compared to laparoscopic surgery. However, since those studies were based on a single-arm trial or retrospective comparison, a clear benefit of robot-assisted gastrectomy has not been demonstrated [19, 20]. A randomized, controlled study (JCOG1907) to confirm the superiority of robot-assisted gastrectomy to laparoscopic gastrectomy in terms of reducing morbidity for clinical T1–2 N0–2 gastric cancer is ongoing. At present, robot-assisted surgery for clinical stage I gastric cancer is weakly recommended. For performing robot-assisted gastrectomy, it is necessary to fulfill the standard quality criteria for the surgeon and the facility (CQ3).

### Reconstruction after gastrectomy

The following reconstruction methods are usually used. Each has advantages and disadvantages. The functional benefits of pouch reconstruction are yet to be established.

#### Total gastrectomy

- Roux-en-Y esophagojejunostomy.
- Jejunal interposition.
- Double-tract method.

#### Distal gastrectomy

- Billroth I gastroduodenostomy.
- Billroth II gastrojejunostomy.
- Roux-en-Y gastrojejunostomy.
- Jejunal interposition.

#### Pylorus-preserving gastrectomy

- Gastro-gastrostomy.

#### Proximal gastrectomy

- Esophagogastrostomy.
- Jejunal interposition.
- Double-tract method.

## Endoscopic resection

### Methods of endoscopic resection

#### Endoscopic mucosal resection (EMR)

The lesion, together with the surrounding mucosa, is lifted by submucosal injection of saline (normo- or hypertonic) and removed using a high-frequency steel snare [21, 22].

#### Endoscopic submucosal dissection (ESD)

The mucosa surrounding the lesion is circumferentially incised using a high-frequency electric knife (usually insulation tipped), and the submucosal layer is dissected from the proper muscle layer [23–25].

### Handling of endoscopically resected specimens

#### Handling of resected specimens

The resected specimens should be handled according to the rules described in the Japanese Classification of Gastric Carcinoma 15th edition [1].

#### Definition of differentiated-type and undifferentiated-type carcinoma

The tumor biopsy specimens and endoscopically resected tumors are histologically classified into either the differentiated type or the undifferentiated type. The former includes malignant epithelial tumor, general type, of papillary adenocarcinoma (pap) and tubular adenocarcinoma (tub1, tub2), and the latter includes that of poorly differentiated adenocarcinoma (por1, por2) and signet ring cell carcinoma (sig) according to the Japanese Classification of Gastric Carcinoma 15th edition.

When mucinous adenocarcinoma (muc) is found at the submucosal layer, the resected specimen is handled as undifferentiated type, regardless of whether it is considered to derive from the differentiated or undifferentiated type.

#### Histological predominance and intratumoral ulcerative findings (UL)

A tumor consisting of components of both differentiated-type and undifferentiated-type carcinoma is, nevertheless, classified into one of the two types according to the quantitative predominance. In addition, when more than one histological type is found in a tumor, all histological types are to be recorded in the order of quantitative predominance, e.g., tub2 > tub1. The diagnosis of UL1 is principally made based on the histological evidence of ulcerative findings. However, the histological diagnosis of UL is sometimes difficult because of a biopsy-derived scar. Thus, endoscopic and/or radiological evidence should also be taken into consideration when making a conclusive diagnosis. A biopsy-derived scar is usually observed histologically as fibrosis restricted to small areas just beneath the muscularis mucosae [26]. If it cannot be discriminated from the ulcer scar, it should be classified as UL1.

### Indication for endoscopic resection (CQ31, 32)

Lesions that could technically be resected by endoscopy are classified into the following three categories depending on the strength of evidence. “A tumor indicated for endoscopic resection as a standard treatment (absolute indication)” is defined as a tumor in which the possibility of harboring lymph node metastasis is less than 1%. For this population, endoscopic resection is expected to have therapeutic effect equivalent to a surgical resection. “A tumor indicated for endoscopic resection as an investigational treatment (expanded indication)” is defined as a tumor for which sufficient evidence for long-term outcome after endoscopic resection is lacking, although the possibility of harboring lymph node metastasis is less than 1%. “A tumor indicated for endoscopic resection as clinical practice under some circumstances (relative indication)” is defined as a tumor which would usually be treated by surgical resection, but for which endoscopic resection may still lead to cure and could, therefore, be an option when surgery cannot be recommended due to various clinical circumstances.

#### Principles for indications

Endoscopic resection is considered for tumors that have a very low possibility of lymph node metastasis and are suitable for en bloc resection [27, 28].

#### Indication

#### Absolute indication

*Absolute indication for EMR or ESD* [29–31].

A differentiated-type adenocarcinoma without ulcerative findings (UL0), in which the depth of invasion is clinically diagnosed as T1a, and the diameter is ≤ 2 cm.

*Absolute indication for ESD*

- A differentiated-type adenocarcinoma without ulcerative findings (UL0), in which the depth of invasion is clinically diagnosed as T1a, and the diameter is > 2 cm.
- A differentiated-type adenocarcinoma with ulcerative findings (UL1), in which the depth of invasion is clinically diagnosed as T1a, and the diameter is ≤ 3 cm.
- An undifferentiated-type adenocarcinoma without ulcerative findings (UL0), in which the depth of invasion is clinically diagnosed as T1a, and the diameter is ≤ 2 cm.

##### Expanded indication [32]

- A locally recurred lesion, in which the depth of invasion is clinically diagnosed as T1a, after initial endoscopic resection of endoscopic curability (eCura) C-1 described below for a lesion with an absolute indication and differentiated-type adenocarcinoma

#### Relative indication

A standard therapy is surgical resection for tumors that do not fulfill the absolute or expanded indications. However, endoscopic resection could be an option for elderly and high-operative-risk patients with severe comorbidities. Such cases are considered relative indications, and endoscopic resection could be performed, provided that consent is obtained from the patient after explaining the risk of residual disease, possibly in the form of lymph node metastasis.

### Curability of endoscopic resection

#### Evaluation of curability

Two factors should be considered for curability assessment: completeness of the primary tumor removal and possibility of lymph node metastasis.

#### Endoscopic curability A (eCuraA)

The resection is classified as endoscopic curability A (eCuraA) when all of the following conditions are fulfilled, provided the cancer has no ulcerative findings (UL0): en bloc resection, any tumor size in case of histologically differentiated type-dominant, tumor size ≤ 2 cm in case of histologically undifferentiated type-dominant, pT1a, negative horizontal margin (HM0), negative vertical margin (VM0), and no lymphovascular infiltration (Ly0, V0). However, if the undifferentiated component of the lesion exceeds 2 cm in length, the endoscopic curability is classified as C-2 (eCuraC-2).

When the cancer has ulcerative findings (UL1), the resection is still classified as eCuraA when all of the following conditions are fulfilled: en bloc resection, tumor size ≤ 3 cm, histologically differentiated type-dominant, pT1a, HM0, VM0, Ly0, V0.

#### Endoscopic curability B (eCuraB)

The resection is classified as endoscopic curability B (eCuraB) for pT1b cancer when all of the following conditions are fulfilled: en bloc resection, histologically of differentiated type-dominant, pT1b1 (SM1) (< 500 μm from the muscularis mucosae), HM0, VM0, Ly0, V0, tumor size ≤ 3 cm. However, if the undifferentiated component is included in the portion of submucosal invasion, the endoscopic curability is classified as C-2 (eCuraC-2) [33].

#### Endoscopic curability C (eCuraC)

The resection is classified as endoscopic curability C (eCuraC) when it does not fulfill the conditions described above to be classified as either eCuraA or eCuraB.

The resection is classified as endoscopic curability C-1 (eCuraC-1) when it is histologically differentiated type either not resected en bloc or had a positive horizontal margin even though fulfilling other criteria to be classified into either eCuraA or eCuraB. All other eCuraC resections are subclassified as endoscopic curability C-2 (eCuraC-2).

### Treatments after endoscopic resection (Fig. 7)

Treatments should be planned as follows after evaluation of curability based on the histological examination of the resected specimens.

**Fig. 7:**
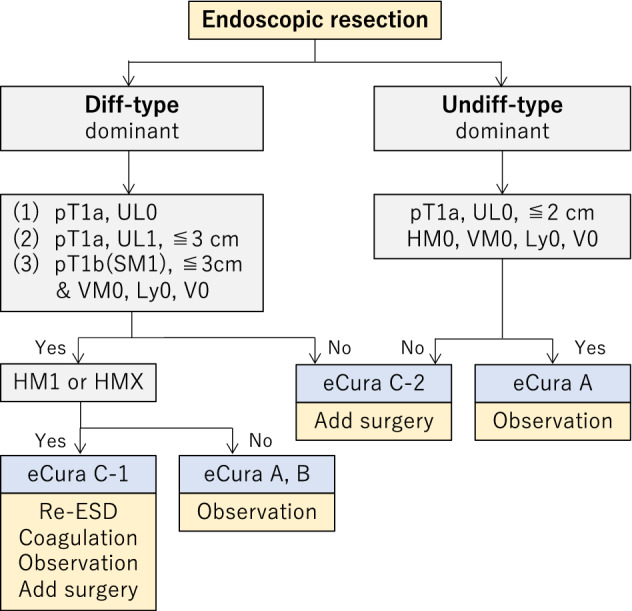
Algorithm showing curability decision and additional treatments for patients who have undergone endoscopic resection

#### Treatments after eCuraA or eCuraB

Follow-up with annual endoscopy is recommended after eCuraA resection [34]. Follow-up with annual or biannual endoscopy and abdominal ultrasonography or computed tomography (CT) for surveillance of metastases is recommended after eCuraB resection [35]. For both eCuraA and eCuraB resections, it has been recommended that examinations for *Helicobacter pylori* be performed, and that if positive, it should be eradicated [36–38]. Since the risk of metachronous gastric cancer is long-lasting, long-term follow-up is required.

#### Treatments after eCuraC-1

Since the risk of harboring lymph node metastasis is low, one of the following alternatives could be selected according to the institutional policy after obtaining the patient’s consent: repeat ESD, surgical resection, close observation expecting a burn effect of the initial ESD, and endoscopic coagulation using a laser or argon-plasma coagulator [39].

When the lesion is differentiated type of ≤ 3 cm and one of UL1, pT1a (M) or pT1b1 (SM1), the size of the residual mucosal lesion should be reassessed by endoscopy. When the sum of the lengths of the resected and residual lesions exceeds 3 cm, gastrectomy with lymphadenectomy should be considered the standard of care. In addition, for patients with a positive horizontal margin within the portion of submucosal invasion and for those who underwent piecemeal resection in which the resection line involved the portion of submucosal invasion, gastrectomy with lymphadenectomy should be recommend, since the histological diagnosis under these circumstances is destined to be uncertain.

#### Treatments after eCuraC-2

Gastrectomy with lymphadenectomy should be considered as the standard of care. When surgery cannot be recommended because of old age or severe comorbidities, the risk of residual disease in the form of lymph node metastasis (Tables 2 [40] and 3 [41]) and the possibility of subsequent local recurrence and/or distant metastasis should be assessed and explained sufficiently to the patients, along with the information that recurrent disease is usually incurable and has a dismal prognosis [42].

**Table 2:**
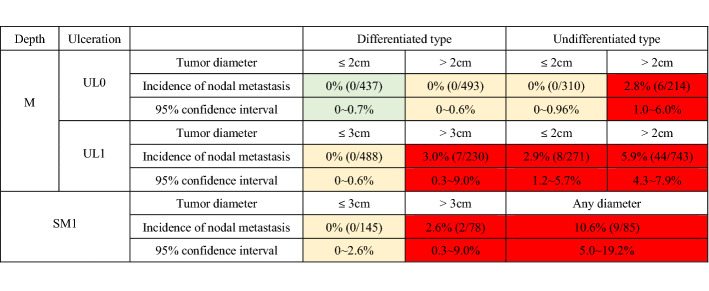
Incidence of nodal metastasis in various categories of early gastric cancer observed from surgically resected specimens operated at National Cancer Center Hospital and Cancer Institute Hospital [34]

Green zone indicates absolute indication for endoscopic resection, yellow zone indicates expanded indication and red zone indicates relative indication

**Table 3:** Incidence of nodal metastasis observed from the specimens of patients who underwent additional gastrectomy with lymphadenectomy after initial treatment with endoscopic resection

Total points	Number of patients (*n* = 1101)	Number of patients with lymph node metastasis (*n* = 94)	Incidence of nodal metastasis (%)	(95% confidence interval)
0	62	1	1.6	(0.0–8.7)
1	341	9	2.6	(1.2–5.0)
2	185	9	4.9	(2.3–9.0)
3	148	11	7.4	(3.8–12.9)
4	132	11	8.3	(4.2–14.4)
5	141	28	19.9	(13.6–27.4)
6	77	21	27.3	(17.7–38.6)
7	15	4	26.7	(7.8–55.1)

Total points refer to the total of following scoring scheme: one point added to each of the following findings: diameter ≥ 3 cm, positive vertical margin, venous invasion, depth ≥ SM2. Three points added to a histopathological finding of lymphatic invasion [35]

## Systemic chemotherapy for unresectable advanced/recurrent gastric cancer (AGC)

Although recent advances in chemotherapy have achieved considerable tumor shrinkage in many cases of AGC, it is difficult to obtain cure by chemotherapy alone. The median survival time achieved in domestic and international clinical trials for the disease at this stage remains about 15 months [43, 44]. The current goal of chemotherapy, therefore, is to delay the manifestation of, or ameliorate, the disease-related symptoms and to prolong survival (Table 4).

**Table 4:** Definition of the evidence level

Strength of the body of evidence	
A (strong)	Strong reliability in the expected value of the effect
B (moderate)	Moderate reliability in the expected value of the effect
C (modest)	Limited reliability in the expected value of the effect
D (weak)	Almost not reliable for the expected value of the effect

Clinical benefits of chemotherapy have been proven in randomized, controlled trials comparing chemotherapy with best supportive care (BSC) in patients with performance status (PS) of 0–2, in terms of overall survival as the primary endpoint [45–47]. Although very rare, some patients with AGC actually survive more than 5 years. Thus, systemic chemotherapy is the treatment to be primarily considered for patients with AGC and those who have undergone non-curative (R2) resection.

## Principles of indications for systemic chemotherapy of AGC

Systemic chemotherapy is indicated for patients with AGC or those who have undergone R2 resection, provided their general condition and major organ functions are preserved. To be more specific, it is indicated for patients having PS 0–2 with either unresectable (locally advanced cancer and/or distant metastases) or recurrent gastric cancer.

### Standard patient criteria for systemic chemotherapy

For administration of chemotherapy, the indication should be decided for each patient by checking the following items.

- Histologically proven gastric cancer
- PS 0–2. Chemotherapy is not generally recommended for patients with PS 3 or worse, and the decision beyond the scope of this recommendation should be made after discreetly considering the safety and clinical consequences for each patient (safety is of a particular concern for AGC with massive ascites or extensive peritoneal metastases).
- Preserved major organ function.
- No serious comorbidities.
- Informed consent obtained from the patient.

#### Routine evaluations before and during chemotherapy

1. The following should be checked or measured prior to initiation of chemotherapy: PS, height, weight, symptoms, physical examination, laboratory data including hepatitis virus tests, and the size of tumor lesions assessed by computed tomography (CT) or other appropriate diagnostic modalities.
2. Response should be assessed by appropriate modalities including CT, gastrointestinal endoscopy, and contrast X-ray examination every 2 or 3 months, comparing the obtained images with those prior to initiation of chemotherapy or at the best response. Tumor response should be evaluated by the Japanese Classification of Gastric Carcinoma or the Response Evaluation Criteria in Solid Tumors (RECIST) to decide whether to continue the ongoing chemotherapy.
3. The decision of whether to continue the ongoing chemotherapy, to modify the drug dosage, or to change the treatment intervals should be made by carefully considering the adverse events and efficacy, and referring to the details of clinical trials showing the clinical significance of the treatment. Cumulative toxicities such as skin toxicities, dysgeusia, and peripheral neuropathy should be considered.
4. Appropriate management is needed for human hepatitis B virus carriers and infected patients to deliver chemotherapy according to the guidelines for reactivation of human hepatitis B virus (ref: https://www.jsh.or.jp/lib/files/medical/guidelines/jsh_guidlines/B_document-3_20200716.pdf, in Japanese).

#### Anti-cancer agents

The following chemotherapeutic agents are proven to be beneficial for AGC: fluorouracil (5-FU), tegafur/5-chloro-2,4-dihydroxypyridine/potassium oxonate (S-1), levofolinate calcium, capecitabine, cisplatin, oxaliplatin, irinotecan, docetaxel, paclitaxel, nab-paclitaxel, trifluridine/tipiracil (FTD/TPI), trastuzumab, ramucirumab, nivolumab, pembrolizumab, and trastuzumab deruxtecan. These agents are used either as monotherapy or combination therapy based on the evidence obtained through clinical trials.

### Definition of the recommendation grade and evidence level for each chemotherapeutic regimen

The recommendation grade for each chemotherapeutic regimen is classified into the following two levels, taking into consideration not only evidence from clinical studies, but also information from clinical practice in Japan.

#### “Recommended regimens”

In this guideline, recommended regimens are defined as those that fulfill one of the following requirements for patients whose general condition is sufficient to meet the inclusion criteria of clinical trials.

- Clinical utility such as superiority over, or non-inferiority in terms of overall survival proven by a domestic or international phase III clinical trial.
- Reproducible clinical benefit for a specific patient population demonstrated by multiple domestic or international clinical trials.
- The regimen recognized as one of the standard regimens that has been adopted as a control arm in multiple domestic or international phase III clinical trials.

#### “Conditionally recommended regimens”

Conditionally recommended regimens are defined as those that fulfill one of the following requirements and could substitute for the “Recommended regimens” when deemed more appropriate after considering factors such as (i) general condition of the patient including disease status, age, organ functions and comorbidities, (ii) social factors such as hospitalization, cost of the treatment, and distance to the hospital, and (iii) personal preference that derives from the type of adverse events.

- Considerable clinical benefit under a specific condition in which the patient may not tolerate the “Recommended regimen”.
- Considerable clinical benefit based on wide usage in Japan in general practice or through interpretation of relevant clinical trials, even though the evidence is not robust enough to be included in the “Recommended regimens”.

**Fig. 8:**
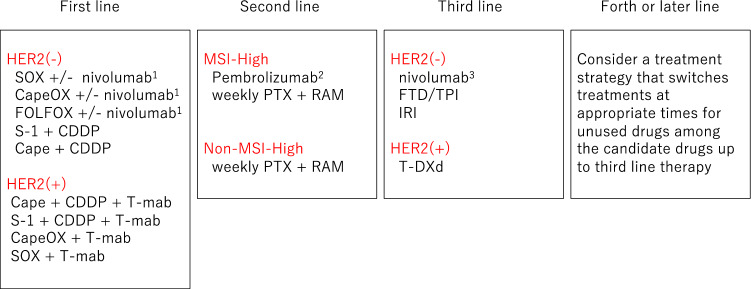
Recommended regimens for the first-, second-, third-, fourth-, or later-line treatments. Only the “Recommended regimens” as defined in the text are included. These regimens are recommended for patients who are in sufficiently good general condition to be eligible for the clinical trials from which the evidence in support of these regimens was generated. 1: Risk–benefit balance of chemotherapy alone and combination with nivolumab should be considered according to the patient’s condition, and either treatment can be selected with the patient’s informed consent. 2: Pembrolizumab in second-line for MSI-High AGC is not recommended when nivolumab was administered in first-line treatment. Weekly paclitaxel plus ramucirumab should be considered in third- or later-line treatment. 3: Nivolumab in third- or later-line treatment is not recommended when pembrolizumab or nivolumab was administered in previous treatment

**Fig. 9:**
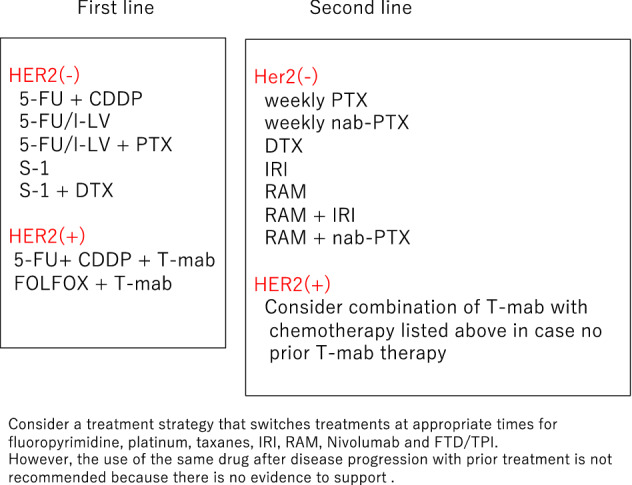
Conditionally recommended regimens shown in alphabetical order. Even when using the Conditionally recommended regimens, refer to Fig. 8 for the basic strategy and attempt to use drugs from all of the following seven categories during the course of the treatment: fluoropyrimidines, platinum, taxanes, irinotecan, ramucirumab, nivolumab, and FTD/TPI. However, it is important to note that continuation of any of the drugs cannot be recommended beyond progression

The “Recommended regimens” and “Conditionally recommended regimens” listed in Figs. 8 and 9 are selected based on voting by the seven medical oncologists who were members of the JGCA Guidelines Committee (the decision required agreement of at least 70% [5 of 7] of the medical oncologists). However, the readers are not necessarily discouraged from using regimens that are not listed in these figures. The selection rule was stringent, and even the regimens that were supported by 50–69% [4 of 7] of the medical oncologists are not listed. Given the complexity of daily clinical practice, there could be situations where regimens that are not listed could, nevertheless, serve as useful options.

Due to the paucity of clinical trial results specific for elderly patients and for patients with impaired organ function or comorbidities, it is not possible to indicate with sufficient evidence which is superior or safer, a “conditionally recommended regimen” delivered at a reduced dosage or modified treatment interval or a “recommended regimen”. Therefore, the optimal therapeutic regimen for these patients should be selected on a case-by-case basis, with the guidelines serving only as a reference. In addition, shared decision-making with patients is important when selecting a treatment method.

### First-line treatment for unresectable advanced/recurrent gastric cancer

Since trastuzumab-containing regimens became the standard of care for HER2-positive gastric cancer, HER2 testing is strongly recommended in all patients planned to receive chemotherapy for unresectable/metastatic gastric cancer. The methods of HER2 testing include immunohistochemistry and in situ hybridization (ISH).

#### HER2-negative gastric cancer

A combination of S-1 and cisplatin (SP) is the standard of care (the recommended regimen) based on the results of two phase III trials conducted in Japan (JCOG 9912 trial [48] and SPIRITS trial [49]). The combination of capecitabine and cisplatin (XP) is recognized as one of the standard treatments in other countries after its non-inferiority to the 5-FU/cisplatin combination (FP) was proven and has been used as a control group in several global phase III studies, such as the ToGA trial [50] and AVAGAST trial [51]. Since the safety and efficacy of XP have been recognized in subset analyses of the Japanese participants in these trials, this combination is added to the list of “Recommended regimens”. CapeOX, a combination of capecitabine and oxaliplatin that was approved in 2014 in Japan, has efficacy that is at least equivalent to the FP shown in the subset analysis of a phase III study (no Japanese patients included) that evaluated triplets that combined these regimens with epirubicin [52]. A combination of S-1 and oxaliplatin (SOX) also demonstrated efficacy similar to SP in the G-SOX study [43]. These oxaliplatin-containing regimens could be delivered more easily than SP or XP because hydration is not required. Furthermore, a combination of 5-FU/ levofolinate calcium (LV) with oxaliplatin (FOLFOX) has been used as a control regimen in the recent comparative studies [53–55] and has been approved in Japan. Thus, FOLFOX can be a useful option. To summarize, the list of “Recommended regimens” for the first-line treatment of unresectable advanced or recurrent gastric cancer (AGC) includes various combinations of fluoropyrimidine and platinum, except for the original FP regimen (Fig. 8).

Three randomized, controlled studies (KEYNOTE-062, ATTRACTION-4, CheckMate 649) investigating the usefulness of immune checkpoint inhibitors in the first-line treatment of AGC (CQ23) were reported [56–58], and nivolumab in combination with chemotherapy (CapeOX, FOLFOX, or SOX) showed better efficacy compared with chemotherapy alone. Therefore, these combinations have become “the Recommended regimen”. However, since a survival benefit of nivolumab in combination with chemotherapy has not been clearly proven for patients with a PD-L1 combined positive score (CPS) less than 5 or unexamined, the risk–benefit balance of chemotherapy alone and in combination with nivolumab should be considered according to the patient’s condition, and either treatment can be selected with the patient’s informed consent (revised from the Japanese version referring to the bullet published in December 2021). AGC patients with insufficient oral ingestion and severe peritoneal metastases (moderate or worse ascites or intestinal stricture), and elderly patients were not specifically investigated in clinical studies. Thus, no standard treatment regimen has been established, but some regimens are conditionally recommended (CQ19).

#### HER2-positive gastric cancer

The definition of HER2-positive in the ToGA trial was either IHC3 + or FISH-positive [50]. In the subgroup analyses of the trial, the survival benefit was more distinct among the IHC3 + or FISH-positive/IHC2 + cohorts. Thus, trastuzumab-containing regimens are currently recommended for patients with IHC3 + or FISH-positive/IHC2 + status in clinical practice. Since continuous infusion of 5-FU is rarely used nowadays, a combination of trastuzumab with XP, which was used in the ToGA study, a combination of trastuzumab with either triweekly or conventional SP, combinations of trastuzumab with either capecitabine plus oxaliplatin (CapeOX) or S-1 plus oxaliplatin (SOX) are the recommended regimens [59–64] because their efficacy was shown in two successive phase II studies consistently.

### Second-line treatment for unresectable advanced/recurrent gastric cancer

Second-line treatment is recommended for patients with sufficient performance status, because several randomized trials demonstrated a significant survival benefit of chemotherapy over best supportive care (BSC), and good outcomes were observed in a phase III trial that compared two chemotherapeutic regimens in the second-line setting. Randomized trials conducted in Germany [65], Korea [66], and the United Kingdom [67] showed a significant survival advantage of second-line chemotherapy (docetaxel or irinotecan) over BSC.

A Japanese phase III trial, WJOG4007, failed to prove the superiority of irinotecan over paclitaxel (weekly administration) in overall survival, but the median survival time was approximately 9 months in both treatment groups, a good outcome when compared with survival data from other trials exploring second-line chemotherapy [68]. Single-agent regimens with one of docetaxel, irinotecan, or paclitaxel (weekly administration), explored in the aforementioned trials, can now be selected as “Conditionally recommended regimens” when the paclitaxel/ramucirumab combination described below is considered unsuitable.

Since the paclitaxel/ramucirumab combination was shown to be superior to weekly paclitaxel monotherapy in a phase III trial (RAINBOW trial) [69], this regimen is currently the “Recommended regimen” (CQ16). In addition, the REGARD trial showed the survival benefit of ramucirumab monotherapy over BSC. Thus, monotherapy using any of the agents including paclitaxel, docetaxel, irinotecan, and ramucirumab is a “Conditionally recommended regimen” when the paclitaxel/ramucirumab combination is deemed unsuitable. Nab-paclitaxel (albumin-conjugated paclitaxel) was approved in Japan in 2013. The clinical trial (ABSOLUTE trial) demonstrated the non-inferiority of weekly administration of nab-paclitaxel over weekly paclitaxel monotherapy [70], and this regimen is also included in the “Conditionally recommended regimens” when ramucirumab is not suitable. A combination of nab-paclitaxel and ramucirumab has also been established and could be used as a “Conditionally recommended regimen” when nab-paclitaxel is preferred over paclitaxel from, for example, the viewpoint of adverse reactions.

The efficacy of continuing trastuzumab beyond progression for HER2-positive gastric cancer initially treated with a trastuzumab-containing regimen has been denied by a randomized trial (WJOG7112G), and it is not recommended (CQ25). At this point, the regimen in second-line treatment recommended for HER-2-positive gastric cancer is the same as that for HER2-negative gastric cancer. Although trastuzumab deruxtecan, which targets HER2, showed efficacy in third- or later-line treatments, its efficacy in second-line treatment will be investigated in the ongoing clinical studies.

The anti-PD-1 antibody pembrolizumab, which is one of the immune checkpoint inhibitors, is reported to be effective for the patient harboring microsatellite instability (MSI)-high gastric cancer. The MSI test is recommended prior to second-line treatment. The frequency of MSI-high in advanced or recurrent gastric cancer is about 3–5%. Pembrolizumab is approved for patients with unresectable advanced or recurrent MSI-high solid tumor that has progressed after chemotherapy. In the sixth version, pembrolizumab monotherapy for gastric cancer with MSI-high is a “Recommended regimen” for second- or later-line treatment for the following reasons: (1) analysis of the KEYNOTE-158 trial, including gastric cancer patients, yielded relatively good response rates and progression-free survival; and (2) pembrolizumab monotherapy showed better results than paclitaxel monotherapy in the subset analyses for MSI-high patients in the KEYNOTE-061 trial, which included Japanese patients. However, no direct comparison between pembrolizumab and paclitaxel plus ramucirumab in the MSI-high papulation has been done. Therefore, it cannot be concluded at this time which is prioritized for patients with MSI-high gastric cancer, paclitaxel plus ramucirumab or pembrolizumab. As of September 2020, immune checkpoint inhibitors are not approved as first-line treatment, but after approval, pembrolizumab monotherapy (in the case of MSI-high) in second-line treatment is recommended only for a case without prior use of immune checkpoint inhibitors.

### Third- or later-line treatment for unresectable advanced/recurrent gastric cancer

Third-line treatment should be considered if good general condition is maintained after the end of second-line treatment, but a careful decision should be made regarding the indications for treatment. Monotherapy with nivolumab, irinotecan, or FTD/TPI is the “Recommended regimen” for third- or later-line treatment for patients with good general condition. In the clinical study in Korea, prolongation of survival by docetaxel or irinotecan was shown in a randomized trial including patients both in second- and third-line treatments, and its subgroup analysis of third-line treatment also suggested a survival benefit of chemotherapy over best supportive care. Irinotecan is currently the mainstream for use in third-line treatment, because paclitaxel plus ramucirumab is recommended as second-line treatment. Nivolumab and FTD/TPI both showed prolonged survival compared with placebo in patients who had prior therapies with more than two regimens (ATTRACTION-2 trial, TAGS trial). Both are “Recommended regimens” for third- or later-line treatment. On the other hand, since no study that directly compared nivolumab, irinotecan, and FTD/TPI was performed, the priority and appropriate treatment sequence are not clear among these three agents.

Treatment with trastuzumab deruxtecan, a drug-conjugated anti-HER2 antibody, showed a significantly higher response rate and longer overall survival than physician-chosen conventional chemotherapy (irinotecan or paclitaxel) in the randomized phase II trial in Asia for HER2-positive gastric cancer patients who were treated with two or more prior regimens. In the DESTINY-Gastric01 trial, the combined alpha-error was set to be less than 0.05 at the two endpoints of response rate and overall survival, and it was below the significance level in the interim analysis. Although the number of cases was small, its survival benefit has been verified statistically. Since trastuzumab deruxtecan is the only drug for which survival prolongation was confirmed in comparison with chemotherapy regimens in third-line treatment, trastuzumab deruxtecan is prioritized for third-line therapy of HER2-positive gastric cancer.

As of September 2020, immune checkpoint inhibitors are not approved as first-line treatment, but after approval, nivolumab monotherapy in third-line treatment is recommended only for a case without prior use of immune checkpoint inhibitors.

## Adjuvant chemotherapy

### Clinical significance of postoperative adjuvant chemotherapy

Postoperative adjuvant chemotherapy is delivered with the intention to reduce recurrence by controlling residual tumor cells following curative resection. As adjuvant chemotherapy, the efficacy of S-1 was proven in 2006 by the ACTS-GC trial [71, 72], a study that secured the place of postoperative S-1 monotherapy as a standard of care. The CLASSIC trial conducted in Korea showed the prolongation of relapse-free survival of capecitabine plus oxaliplatin therapy (CapeOx) for TNM Stage II/III gastric cancer, and a study conducted in Japan confirmed its safety in Japanese patients [73]. A combination of S-1 and docetaxel was shown to have significant benefit in relapse-free survival over S-1 alone for Stage III gastric cancer in the JACCRO GC-07 trial [74]. After the systematic review for the preparation of this guideline was completed (December 2020), it was reported that S-1 plus oxaliplatin (SOX) therapy significantly prolonged disease-free survival, which is the primary endpoint, compared with S-1 in the phase III ARTIST2 trial of adjuvant chemotherapy for postoperative stage (pStage) II/III with D2 dissection [75]. Therefore, adjuvant chemotherapy with S-1 alone/combination therapy for pStage II/III gastric cancer is recommended because it was shown to improve survival after curative resection.

### Indications

One-year administration of S-1 for pStage II gastric cancer showed good clinical results (3-year relapse-free survival rate 93.1%, 3-year overall survival rate 96.1%) in the JCOG 1104 trial [76]. Together with the results of the ACTS-GC trial, 1-year postoperative adjuvant chemotherapy with S-1 is recommended for pStage II gastric cancer. On the other hand, S-1 monotherapy is a conditionally recommended regimen for pStage III gastric cancer, because combination therapies are recommended based on the results of the JACCRO-GC07 trial. Since S-1 plus docetaxel and capecitabine plus oxaliplatin have not been directly compared among combination regimens, it is not possible to conclude which of these combination therapies is more effective at this time. It is important to select an appropriate regimen and maintain an appropriate dose and schedule, taking into consideration not only the pathological findings, but also the patient’s general condition and the occurrence of adverse events.

Postoperative chemotherapy for curatively resected Stage IV gastric cancer is weakly recommended because its effectiveness has been suggested, but there is no evidence based on a comparative clinical study (CQ29).

Several studies showed consistent results that chemotherapy for gastric cancer of CY1 after gastrectomy leading to macroscopically no residual tumor except for CY1 can achieve a 5-year survival rate of around 25%, although this is not strictly adjuvant chemotherapy (CQ30).

## Neoadjuvant chemotherapy

Neoadjuvant chemotherapy is premised on “curative resection” based on diagnostic imaging. It should be strictly distinguished from chemotherapy followed by surgery for borderline resectable cases and for initially unresectable cases who are converted to resectable due to its significant effect.

Though a wealth of experience in postoperative adjuvant chemotherapy has been accumulated in Japan, there are still not a few cases for whom it is difficult to perform intensive adjuvant chemotherapy due to decreased oral intake and complications. On the other hand, the curative rate is expected to be improved by neoadjuvant chemotherapy, because it has the advantage of delivering intensive chemotherapy before surgery. However, since postoperative adjuvant chemotherapy targets patients who have undergone curative resection, the indication can be accurately determined based on histological findings, whereas the indication for preoperative adjuvant chemotherapy is determined based on diagnostic imaging. There is a disadvantage of neoadjuvant chemotherapy that some overdiagnosed cases who actually have early cancer not requiring peri-operative chemotherapy and underdiagnosed cases who actually have unresectable peritoneal metastases not detected by conventional imaging examination can be included as targets of neoadjuvant chemotherapy. There are also disadvantages such as the risk of being unresectable due to progression during chemotherapy and increased postoperative complications.

Recommendation for neoadjuvant chemotherapy should be made after carefully weighing these disadvantages against advantages. Thus, safety issues in terms of adverse events during chemotherapy and incidence of postoperative morbidity, accuracy of pretreatment staging, incidence of unresectable disease due to progression during neoadjuvant treatment, and QOL should be analyzed in future neoadjuvant trials in addition to proving superiority in survival outcomes over standard postoperative adjuvant therapies.

Neoadjuvant chemotherapy has been a standard of care in Western countries, and the superiority of neoadjuvant chemotherapy has been reported in Chinese and Korean clinical trials [77, 78]. In Japan, good results have been reported with neoadjuvant chemotherapy with S-1 plus cisplatin combination therapy for “bulky N”, and it is recognized as standard treatment. However, no superiority of neoadjuvant chemotherapy with S-1 plus cisplatin was shown for scirrhous gastric cancer with a poor prognosis [79].

Taken together, although the effectiveness of neoadjuvant chemotherapy has been consistently shown in reports from other countries, various problems have been noted, and there is no consensus about introducing it into clinical practice in Japan (CQ28).

## Palliative care

Palliative care is an approach that improves the QOL of patients and their families facing the problems associated with life-threatening illness through the prevention and relief of suffering by means of early identification and careful assessment and treatment of pain and other problems, physical, psychosocial, and spiritual (WHO Definition of Palliative Care, 2002) [80]. The European Society for Medical Oncology defined ‘Supportive care’ as care that aims to optimize the comfort, function, and social support of the patients and their family at all stages of the illness, and ‘Palliative care’ as care when cure is not possible [81]. In Japan, supportive care is defined as ‘prevention, treatment, and care to reduce symptoms caused by cancer itself and side effects, complications, and sequela associated with cancer treatment’ in the Basic Plan for Promotion of Cancer Control. Therefore, the definition of “palliative care and supportive care” is not the same inside and outside Japan. In addition, there is a large overlap between them, and it is appropriate to comprehensively consider it as “support/palliative medicine”. This guideline sixth edition describes gastrointestinal stent placement (CQ26) and cell-free and concentrated ascites reinfusion therapy (CART) (CQ27), which are characteristically performed as supportive and palliative care for gastric cancer patients. Gastric cancer patients and their families also have various mental, social, and spiritual pains, in addition to physical pains like other cancers. Palliative and supportive care for pain common to all these cancer treatments plays a basic part in cancer medical treatment. Many clinical studies are being conducted in the field of palliative and supportive care, mainly on pain management. Readers of this guideline need to learn about palliative treatment for pain, communication technology, and symptom management, in addition to the clinical questions.

## Clinical pathway after surgery for gastric cancer

The enhanced recovery after surgery (ERAS) protocol is also widely used in gastric cancer, and its usefulness has been evaluated (CQ16) [82]. The introduction of ERAS has been shown to enable early discharge. However, increased complications by accelerating the timing of oral intake have been reported [83], and it is necessary to consider the optimal timing at each facility. If there are no particular problems with the patient getting out of bed, it may be possible to initiate oral fluid intake on or after postoperative day 1, initiate solid food intake on or after day 2, and discharge on postoperative days 7–10 (Table 5).

**Table 5:** A common clinical pathway for distal, total, and proximal gastrectomy

Clinical items	Day of the clinical pathway
Removal of nasogastric tube	Before or on postoperative day 1
Initiation of oral fluid intake	On or after postoperative day 1
Initiation of solid food intake	Postoperative days 2–4
Prophylactic administration of antibiotics	Only on the day of operation
Removal of epidural tube	Before or on postoperative day 3
Removal of urinary catheter	Before or on postoperative day 3
Intravenous fluid administration	Until postoperative days 5–7
Removal of intra-abdominal drains	Before or on postoperative day 5
Discharge from the hospital	Postoperative days 8–14

## Follow-up surveillance after surgery for gastric cancer (CQ17)

**Fig. 10:**
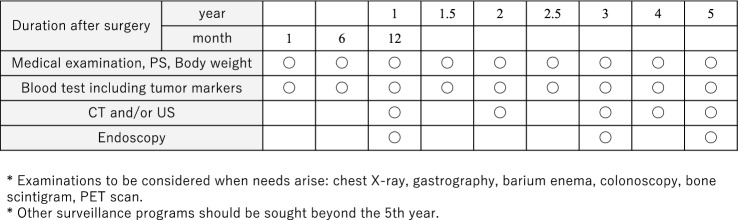
Postoperative follow-up for Stage I gastric cancer patients

**Fig. 11:**
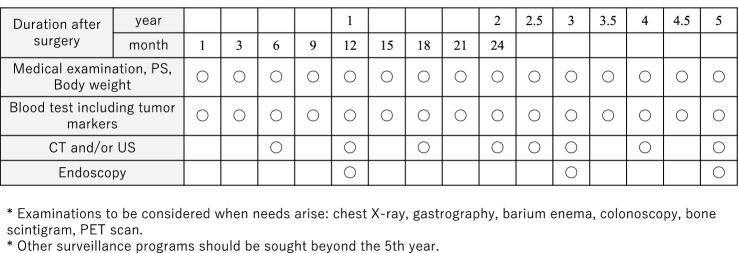
Postoperative follow-up for Stage II–III gastric cancer patients

Life guidance after gastrectomy and treatment for post-gastrectomy syndrome are provided, and follow-up is systematically performed according to the risk of recurrence for early detection of recurrence and secondary cancer. There is little evidence that it can be expected to prolong survival (CQ17) [84, 85]. In addition, since there is no prospective research on regular postoperative follow-up methods, there is little evidence of appropriate follow-up test and follow-up intervals. However, CT, tumor markers (CEA + CA19-9), and endoscopy are thought to be useful to detect recurrence, remnant tumor, and double cancer judging from some retrospective studies. Tumor markers are elevated at the time of recurrence and may precede diagnostic imaging findings by about 2 or 3 months [86]. Based on the results of recurrence/relapse time, follow-up as shown in Fig. 10 for early-stage cancer and Fig. 11 for advanced cancer is presented for reference.

In principle, follow-up for 5 years after surgery is required. However, since recurrence or metachronous multiple cancer may be discovered after 5 years [87], users should individually decide the effective use of not only the users’ own facility, but also referring doctors, collaborating doctors, basic medical examinations, workplace examinations, and comprehensive medical checkups. The contribution of the planned follow-up after surgery to patients’ survival should be scientifically validated in the future.

## Clinical questions for surgery

### *CQ1* Is laparoscopic gastrectomy recommended for cStage I gastric cancer?

#### Recommendation

Laparoscopic distal gastrectomy for cStage I gastric cancer is strongly recommended as one of the standard treatments (consensus rate 100%, 8/8, strength of evidence A). Laparoscopic total gastrectomy or proximal gastrectomy is weakly recommended (consensus rate 100%, 8/8, strength of evidence C). All surgical procedures must be conducted by a qualified surgeon in the endoscopic surgical skill qualification system of the Japanese Society of Endoscopic Surgery or a surgeon with equivalent skills or under the guidance of an instructor with equivalent skills.

### *CQ2* Is laparoscopic gastrectomy recommended for cStage II/III gastric cancer?

#### Recommendation

Clear recommendations cannot be provided for laparoscopic surgery for cStage II/III gastric cancer. (consensus rate 71.4%, 5/7, strength of evidence C).

### *CQ3* Is robot-assisted surgery recommended for gastric cancer?

#### Recommendation

Robot-assisted surgery is weakly recommended for cStage I gastric cancer (consensus rate 100%, 8/8, strength of evidence C). The procedures must be conducted by a qualified surgeon in the endoscopic surgical skill qualification system of the Japanese Society of Endoscopic Surgery and is proficient in this surgery, or by a board-certified surgeon in gastroenterology under the guidance of a proctor certified by the Japanese Society of Endoscopic Surgery. All these procedures must be performed in a certified facility.

### *CQ4* Is pylorus-preserving gastrectomy (PPG) recommended for early gastric cancer in the middle portion of the stomach?

#### Recommendation

PPG is weakly recommended for early gastric cancer in the middle portion of the stomach (consensus rate 100%, 8/8, strength of evidence C).

### *CQ5* Is proximal gastrectomy recommended for early proximal gastric cancer?

#### Recommendation

Proximal gastrectomy is weakly recommended for early proximal gastric cancer (consensus rate 100%, 8/8, strength of evidence C).

### *CQ6* Is omentectomy recommended for advanced gastric cancer?

#### Recommendation

Omentectomy is weakly recommended for cT3–T4 gastric cancer (consensus rate 100%, 8/8, strength of evidence C).

### *CQ7* Is splenic hilar lymph nodes dissection recommended for advanced proximal gastric cancer?

#### Recommendation

It is strongly recommended not to perform splenectomy or splenic hilar lymph node dissection for tumors without greater curvature invasion (consensus rate 100%, 8/8, strength of evidence A). Splenectomy or splenic hilar lymph node dissection is weakly recommended for tumors with greater curvature invasion (consensus rate 87.5%, 7/8, strength of evidence C).

### *CQ8* Is PET-CT scan recommended for the staging of gastric cancer?

#### Recommendation

Not performing PET-CT scan for the staging of gastric cancer is weakly recommended (consensus rate 100%, 8/8, strength of evidence C).

### *CQ9* Is staging laparoscopy recommended for the decision regarding treatment strategy in advanced gastric cancer?

#### Recommendation

Staging laparoscopy is weakly recommended to determine the treatment strategy for patients with advanced gastric cancer who are likely to have peritoneal metastasis (consensus rate 100%, 8/8, strength of evidence C).

### *CQ10* Is surgical treatment for oligo metastases recommended?

#### Recommendation

Surgical resection after neoadjuvant chemotherapy is weakly recommended for a small number of paraaortic lymph node metastases confined to No.16a2/b1. In addition, surgical resection is weakly recommended for solitary liver metastasis without other incurable factors (consensus rate 100%, 7/7, strength of evidence C).

### *CQ11* Is conversion surgery recommended?

#### Recommendation

Conversion surgery for patients with stage IV gastric cancer is weakly recommended with the condition that chemotherapy provides a certain antitumor effect, the response is maintained, and R0 resection is possible (consensus rate 100%, 7/7, strength of evidence D).

### *CQ12* Is mediastinal lymph node dissection recommended for the surgery for esophagogastric junctional cancer?

#### Recommendation

In surgery for esophagogastric junctional cancer deeper than cT2, lower mediastinal lymph node dissection is weakly recommended if the esophageal invasion length is more than 2 cm, and upper, middle, and lower mediastinal lymph node dissection is weakly recommended if the esophageal invasion length is greater than 4 cm (consensus rate 100%, 9/9, strength of evidence C).

### *CQ13* Is para-aortic lymph node (No.16a2lat) dissection recommended for esophagogastric junctional cancer?

#### Recommendation

No clear recommendations are available for para-aortic lymph node (No.16a2lat) dissection in the surgery for esophagogastric junctional cancer (no consensus was reached by two votes. Strength of evidence C).

### *CQ14* Is proximal gastrectomy recommended for esophagogastric junctional cancer?

#### Recommendation

Proximal gastrectomy is weakly recommended for esophagogastric junctional cancer (consensus rate 100%, 9/9, strength of evidence C).

### *CQ15* Is lymph node dissection with splenectomy recommended for carcinoma of the gastric remnant?

#### Recommendation

Splenic hilar dissection with splenectomy is weakly recommended for advanced carcinoma of the gastric remnant with greater curvature invasion (consensus rate 100%, 6/6, strength of evidence D). Not performing splenic hilar lymph node dissection with splenectomy for tumors without greater curvature invasion is weakly recommended (consensus rate 100%, 6/6, strength of evidence D).

### *CQ16* Is the enhanced recovery after surgery (ERAS) protocol recommended for perioperative management of gastric cancer?

#### Recommendation

The ERAS protocol is strongly recommended for perioperative management of gastric cancer (consensus rate 100%, 8/8, strength of evidence A).

### *CQ17* Is systematic follow-up after surgery recommended?

#### Recommendation

From the viewpoint of early detection of recurrence and improved survival, the usefulness of systematic follow-up after radical gastrectomy has not been demonstrated. However, systematic follow-up is weakly recommended because prolongation of the survival period is expected in cases in which post-recurrence treatment is effective, along with medical guidance after gastrectomy and treatment for post-gastrectomy syndrome (consensus rate 100%, 8/8, strength of evidence D).

## Clinical questions for chemotherapy

### Clinical questions regarding chemotherapy for unresectable advanced/recurrent gastric cancer (AGC)

### *CQ18* Is chemotherapy recommended for an elderly patient with AGC?

#### Recommendation

Chemotherapy is strongly recommended for a fit elderly patient, based on a discreet assessment of the general condition (consensus rate 100%, 4/4, strength of evidence B). Otherwise (vulnerable/unfit), no clear recommendations are made because the situation varies (consensus rate 100%, 4/4, strength of evidence B).

### *CQ19* Is chemotherapy recommended for patients with impaired oral intake or massive ascites due to extensive peritoneal disease?

#### Recommendation

Chemotherapy is weakly recommended for patients with impaired oral intake or massive ascites after careful assessment of the general condition (consensus rate 100%, 5/5, strength of evidence C).

### *CQ20* Is chemotherapy recommended for patients with bone marrow carcinomatosis?

#### Recommendation

Chemotherapy is weakly recommended for patients with bone marrow carcinomatosis (consensus rate 100%, 5/5, strength of evidence D).

### *CQ21* Is chemotherapy recommended for patients with metastases of central nervous system?

#### Recommendation

Chemotherapy is weakly recommended for patients with metastases of the central nervous system who are in good general condition (consensus rate 100%, 5/5, strength of evidence D).

### *CQ22* Is personalized medicine based on genomic profiling test recommended for AGC?

#### Recommendation

It is weakly recommended to treat a previously-treated AGC patient based on the genetic alterations obtained from genomic profiling tests (consensus rate 100%, 5/5, strength of evidence C).

### *CQ23* Are immune checkpoint inhibitors for the first-line treatment of AGC recommended?

#### Recommendation

The original version of the sixth edition refrained from recommending the use of immune checkpoint inhibitors since they had not been approved in Japan as of September 2020 (consensus rate 100%, 7/7, strength of evidence B). After the approval in December 2021, a bulletin was issued by the guideline committee, strongly recommending the use of nivolumab among patients with CPS ≥ 5 status while requesting discreet decisions for patients with CPS of less than 5.

### *CQ24* Are combination therapies of fluoropyrimidine and platinum recommended for recurrence after perioperative chemotherapy?

#### Recommendation

Combination therapies of fluoropyrimidine and platinum for recurrence later than six months after adjuvant chemotherapy are weakly recommended (consensus rate 100%, 5/5, strength of evidence C).

### *CQ25* Is continued drug use beyond disease progression recommended for AGC?

#### Recommendation

It is strongly recommended not to continuously use S-1 and trastuzumab after disease progression of AGC (consensus rate 100%, 5/5, strength of evidence B).

### *CQ26* Is gastrointestinal stent placement recommended for palliative treatment of advanced gastric cancer?

#### Recommendation

Gastrojejunostomy or gastrointestinal stent placement for impaired oral ingestion by gastric outflow tract obstruction (gastric outer obstruction) due to gastric cancer is weakly recommended (consensus rate 100%, 5/5, strength of evidence C).

### *CQ27* Is cell-free and concentrated ascites reinfusion therapy (CART) recommended for palliative treatment of advanced gastric cancer?

#### Recommendation

There is no clear recommendation regarding CART as palliative treatment for advanced gastric cancer with ascites. For its implementation, it is necessary to consider the indication taking into account facility equipment status and patient background. Abdominal puncture drainage is used to improve the symptoms of patients suffering from abdominal fullness due to ascites (consensus rate 80%, 4/5, strength of evidence D).

## Clinical questions for perioperative chemotherapy

### *CQ28* Is neoadjuvant chemotherapy for curatively resectable advanced gastric and esophagogastric junctional cancer recommended?

#### Recommendation

There is no clear recommendation for neoadjuvant chemotherapy for curatively resectable advanced gastric and esophagogastric junctional cancer (consensus rate 71.4%, 5/7, strength of evidence B).

### *CQ29* Is adjuvant chemotherapy recommended for stage IV gastric cancer with R0 resection?

#### Recommendation

Adjuvant chemotherapy is weakly recommended for stage IV gastric cancer with R0 resection (consensus rate 100%, 7/7, strength of evidence C).

### *CQ30* Is combination therapy of fluoropyrimidine and platinum recommended for resected gastric cancer of CY1?

#### Recommendation

Not using combination therapy of fluoropyrimidine and platinum for resected gastric cancer of CY1 is weakly recommended (consensus rate 100%, 7/7, strength of evidence C). S-1 monotherapy is weakly recommended for resected gastric cancer of CY1 (consensus rate 100%, 7/7, strength of evidence C).

## Clinical questions for endoscopic resection

### *CQ31* Is endoscopic resection recommended for elderly patients?

#### Recommendation

Endoscopic resection is strongly recommended for elderly patients, paying attention to the risk of complications associated with treatment (especially pneumonia) (consensus rate 100%, 10/10, strength of evidence C).

### *CQ32* Is endoscopic resection recommended for patients being treated with antithrombotic drugs?

#### Recommendation

Endoscopic resection is strongly recommended for patients being treated with antithrombotic drugs, carefully considering the benefits and disadvantages associated with treatment (consensus rate 89%, 8/9, strength of evidence C).
